# PI3K/Akt Signaling Pathway Modulates Influenza Virus Induced Mouse Alveolar Macrophage Polarization to M1/M2b

**DOI:** 10.1371/journal.pone.0104506

**Published:** 2014-08-08

**Authors:** Xiangfeng Zhao, Jianping Dai, Xuejun Xiao, Liqi Wu, Jun Zeng, Jiangtao Sheng, Jinghua Su, Xiaoxuan Chen, Gefei Wang, Kangsheng Li

**Affiliations:** 1 Department of Microbiology and Immunology, Shantou University Medical College, College, Shantou, Guangdong, China; 2 Department of Nursing, Guilin Medical University, Guilin, Guangxi, China; University of Hong Kong, Hong Kong

## Abstract

Macrophages polarized to M1 (pro-inflammation) or M2 (anti-inflammation) phenotypes in response to environmental signals. In this study, we examined the polarization of alveolar macrophage (AM), following induction by different influenza virus strains (ST169 (H1N1), ST602 (H3N2) and HKG9 (H9N2)). Macrophages from other tissues or cell line exert alternative responding pattern, and AM is necessary for investigating the respiratory system. AM polarized toward the M1 phenotype after 4 hours of infection by all three virus strains, and AM to presented M2b phenotype after 8 hours induction, and immunosuppressive phenotype after 24 hours of induction. Protein expression assay showed similar results as the gene expression analysis for phenotype verification. The ELISA assay showed that TNF-α secretion was up-regulated after 4 and 8 hours of infection by influenza viruses, and it returned to basal levels after 24 hours of infection. IL-10 expression was elevated after 8 and 24 hours of infection. Immunofluorescence showed that iNOS expression was up-regulated but not Arg1 expression. Influenza virus notably increased phospho-Akt but not phospho-Erk1/2 or phospho-p38, and the AM polarization pattern have been changed by LY294002 (PI3K inhibitor). In conclusion, our results demonstrate the dynamic polarization of AM induced by influenza viruses, and suggested that PI3K/Akt signaling pathway modulates AM polarization to M1/M2b.

## Introduction

Influenza virus is a highly contagious RAN virus causing infection of the upper and lower respiratory tract, making influenza a leading cause of human morbidity and mortality worldwide. Primary viral pneumonia is the most severe complication observed during influenza infection and reveals high mortality [1]. Influenza virus causes pathological damages in host tissues by initial viral replication in bronchial and alveolar epithelial cells followed by infections spreading to adjacent resident alveolar macrophages (AM) [2]. Influenza virus infection triggers strong inflammatory responses that can result in fatal pneumonia. During influenza virus infection, lung macrophages involved innate immune system is mobilized to participate into those inflammatory responses. Lung macrophages can be subdivided into alveolar, pleural, interstitial and intravascular macrophages, with AM considered as the most important cell of the innate immune system [3]. AM phagocytose virus particles and apoptotic cells to protect the lungs from influenza-induced damage [4], [5], [6]. Existing results confirmed that depletion of AM lead to enhanced virus replication and more severe disease progress during influenza virus infection [7], suggesting an indispensable role of AM in anti-influenza virus immune responses.

Macrophages exert various biological functions in a broad spectrum of acute and chronic inflammatory conditions. Macrophages undergo highly reversible and transient polarization processes in response to comprehensive environmental signals [8], [9], [10]. For the sake of simplicity and efficiency, a model system has been established to clarify macrophages on a continuum with pro-inflammatory M1 macrophages representing one extreme, and anti-inflammatory M2 macrophages subdivided into M2a, M2b and M2c, representing the opposite extreme. M1 and M2 macrophages can serve distinct functions in the regulation of the inflammatory response [11], [12]. M1 macrophages are critical effector cells that kill microorganisms and thus benefit the host, albeit this pro-inflammatory activity must be carefully titrated. In contrast, M2 macrophages are involved in the resolution of inflammation [8]. M2 cells are generally characterized by a low level of production of pro-inflammatory cytokines, while M2b cells are an exception for the retaining high levels of inflammatory cytokine production with concomitant secretion of high IL-10 and low IL-12 [9], [13].

Macrophages could initiate polarization process on stimulation by microbe infection, and polarized macrophages could counteract on surrounding milieu via altered phenotypes. Pro-inflammatory M1 cells play a key role in virus clearance, but excess inflammation is harmful to the lung [14]. On the contrary, M2 (anti-inflammation) cells play a key role in protecting the lung. The pro-inflammatory response from an influenza infection must be balanced by regulatory and inhibitory effector mechanisms to protect bystander tissue damage from the effects of excess inflammation, promote host tissue repair after viral clearance and preserve oxygenation [15], [16]. John R. Teijaro and colleagues demonstrated that suppression of early innate immune responses resulted in reduced mortality during infection with a human pathogenic strain of influenza virus [17], verifying that the balance of M1/M2 in AM is important for lung protection. Classification of the function of M1 and M2 macrophages have provided an important tool for understanding the regulation of the inflammatory process, however, the underlying mechanisms are still largely unknown.

AM plays important roles in protecting the lungs from influenza viruses infection. AM is essential for controlling H1N1 influenza viruses in pigs [18], and AM depletion before a sublethal infection with 1918 H1N1 virus resulted in uncontrolled virus growth and mortality in mice [19]. In addition, AM depletion was associated with decreased expression of cytokines and chemokines. Differential polarization of M1/M2 macrophages showed different roles in protecting lung tissues through cytokine or chemokine release and cellular signal transduction. During viral infections, macrophage polarization must be balanced to avoid excessive inflammatory responses and protect the lungs against invading pathogens [20]. Until now, influenza virus induced AM activation featured as direct virus phagocytes, cytokine releasing and immunoregulation have been widely investigated [18], [21], [22]. Up to date, the roles of influenza virus in AM polarization are still unclear and the molecular mechanisms that govern M1/M2 polarization induced by influenza virus remain a conundrum.

In this study, we investigated the polarization patterns of mouse AM and pertinent correlation with infection of human influenza virus strains. To this end, global transcriptional profiles were used to assess the M1 and M2 polarization-related genes in AM. In this study, we collected AM from mouse bronchoalveolar lavage (BAL) and activated the cells in vitro with different subtypes of influenza virus (human influenza virus strains ST169 (H1N1) and ST602 (H3N2) and avian influenza virus strain HKG9 (H9N2)). After 4, 8 or 24 hours of infection, we examined the expression of polarization-markers to exhibit the polarization pattern of AM as well as potential molecular mechanism involved in this process.

## Materials and Methods

### Ethics statement

This study was preapproved by the Ethical Committee of Shantou University Medical College and was conducted in compliance with the Experimental Animal Management Bill issued on 14th November 1988 (Decree No. 2 of National Science and Technology Commission, China), and the National Institute of Health Guide for the Care and Use of Laboratory Animals (NIH Publications No. 80-23, revised 1996).

### Animal, reagents and virus

Eight-week old female C57BL/6 mice were purchased from Shantou University Medical College Laboratory Animal Center, Shantou, Guangdong, China. Animals were housed in specific pathogen-free level facilities. The mice were housed with five mice per standard laboratory cage (26 cm×42 cm×15 cm) in a constant temperature and humidity vivarium (12-h light, 12-h dark cycle). The mice were adapted to the environment before any behavioral experiments were performed. Raw264.7 was maintained in our laboratory. Culture medium for the generation of mouse alveolar macrophages consisted of RPMI 1640 medium (Gibco BRL, NY, NY, USA), supplemented with 2 mM L-glutamine, 100 U/ml penicillin, 100 mg/ml streptomycin and 10% heat inactivated fetal calf serum. The influenza virus strains ST169 (A/Shantou/169/06, H1N1), ST602 (A/Shantou/602/06, H3N2) and HKG9 (A/Chicken/Hong Kong/G9/97, H9N2) were maintained in our laboratory.

### Cell preparation and alveolar macrophage culture

AM was purified according to the method of Small CL et al. [23]. Briefly, anesthetized mice were sacrificed, the lungs from 36 mice were lavaged with RPMI 1640 contain 10% FCS, and all BAL were mixed in a 50 ml tube. BAL fluids were then plated in 36 dishes (35 mm diameter), and the AM was allowed to adhere for 2 hours at 37°C, 5% CO_2_.

The quantity and purity of the AM was detected as follow: BAL cells were incubated for 30 minutes at 4°C with rat-anti-F4/80 monoclonal antibody (mAb) conjugated to Alexa Fluor 488 (Invitrogen, Carlsbad, CA, USA). Then, the cells were analyzed using a FACS Calibur flow cytometer (BD, San Jose, CA, USA). Flow cytometry was also processed to detect the purity of adherent cells, briefly, the BAL cells were seeded on plates for 2 hours and then non-adherent cells were washed, the adherent cells were scraped and fixed with 4% paraformaldehyde at 4°C for 30 minutes, and then incubated for 2 hours with a 1∶300 dilution of the F4/80 mAb labeled with Alexa Fluor 488 for flow cytometry analysis.

Immunofluorescence was processed also to determine the purity of the AM, we seeded BAL cells directly on coverslips. After 2 hours, non-adherent cells were washed and the cells on the coverslips were fixed with 4% paraformaldehyde at 4°C for 30 minutes. After a washing step using PBS, the cells were incubated for 2 hours with a 1∶300 dilution of the F4/80 mAb labeled with Alexa Fluor 488 at 4°C. After three 5 minutes washing steps with PBS, the cells were counterstained with Hoechst (1∶300 in PBS for 15 minutes). Finally, after a final 5 minutes washing step in PBS, the cells were covered with a glass slide. The purity of the macrophages was determined using a fluorescent microscope (Nikon, Tokyo, Japan) at room temperature; fluorescent images are at a 400× final magnification.

To confirm if AM is replaceable in this study, we collected different macrophages to detect their response toward influenza virus infection, including AM, BMDM (bow marrow derived macrophage), PM (peritoneal macrophage) and Raw264.7 cell line; the cells were collected by the described method [24], [25], [26] and were subsequently infected with ST169 (H1N1) for 8 hours by the method described below.

### In vitro stimulation of alveolar macrophages

Influenza viruses were propagated and plaque forming units (PFU) have been detected as described in our previous study [27], [28]. PFU = ST169 (1.5×10^9^), ST602 (4.5×10^9^) and HKG9 (0.8×10^9^). Before the experiments of in vitro stimulation of AM, preliminary experiment was processed for optimizing the efficient but non-cytotoxicity MOI to the macrophages. AM was purified as described, washed with RPMI 1640 to remove FCS, infected with influenza virus at a multiplicity of infection (MOI) of 2 in serum-free RPMI 1640 and incubated at 37°C, 5% CO_2_. AM was harvested after 4 hours, 8 hours and 24 hours. The negative control (the same volume of influenza virus culture medium, MEM containing 0.5 µg/ml TPCK-trypsin and 2% BSA fraction V, was added instead of virus) were harvested follow the same process. The supernatants were also harvested and stored at −20°C for using in an ELISA assay.

### Analysis of gene expression by qRT-PCR

Total RNA was isolated from AM using Trizol reagent (Invitrogen) according to the manufacturer's instructions. Total RNA from the AM was used for first-strand cDNA synthesis using cDNA First Strand Synthesis Kit (Invitrogen) following the manufacturer's instructions.

By qRT-PCR, the relative gene expression of M1 associated markers (STAT1, TNF-α, MCP1, IL-6, iNOS and IL-12), M2 associated markers (STAT6, ARG-1, MGL-1, CD163, and IL-10), and innate immune receptors (TLR2, TLR4, TLR5 and TLR6), as well as GAPDH as a control, were quantified. The primers were listed in Table S1 in File S1. Using ABI Prism 7300 Sequence Detection System, PCR reactions were set up in a 20-µl reaction volume. The qRT-PCR reactions were performed using a Fast Start Universal SYBR Green Master kit (Roche, Mannheim, BW, Germany) and the ABI Prism 7300 qRT-PCR system with GAPDH as a reference control. The 2^−ΔΔct^ method was used to analyze the results of the qRT-PCR [29].

### ELISA analysis of TNF-α and IL-10 expression

AM culture medium supernatants were stored at −20°C until analyzed by sandwich ELISA. Supernatants were thawed on ice, and the sandwich ELISA was performed according to the instructions in the ELISA kit (Neobioscience, Shenzhen, Guangdong, China).

### Immunofluorescence analysis of iNOS and Arg1 expression

AM was seeded in a Lab-Tek Chamber Slide System (Nalge Nunc, Naperville, IL, USA), infected with viruses at a MOI of 2, and then treated as described above. Samples were blocked with serum and then exposed to rabbit polyclonal iNOS antibody (Genetex, San Antonio, Texas, USA) at a 1∶100 dilution as a marker of M1 polarization and a goat polyclonal Arg1 antibody (Abcam, Cambridge, UK) at 1∶100 as a marker of M2 polarization. Samples were treated with Cy3 conjugated donkey anti-goat IgG (Beyotime, Hangzhou, China) followed by Alexa Fluor 488 conjugated goat-anti-rabbit IgG secondary antibody (Beyotime). Three images were obtained randomly per slide to determine iNOS and Arg1 expression. Fluorescence microscope (Nikon 90i) was used to image the fluorescent staining. All fluorescent images are at a 1000× final magnification.

### Western blotting

Cell lysates were suspended in (1∶4; v/v) educing sample buffer, boiled, and separated by SDS-PAGE. Proteins were transferred onto PVDF membranes with a semi-dry blotting appa-ratus (Bio-Rad Laboratories). Membranes were blocked with BSA and incubated overnight at 4°C with the following antibodies: Anti-pAkt, anti-Akt, anti-pErk, anti-Erk, anti-p-p38 and anti-p38 antibodies (Cell Signaling Technology), and subsequently with the appropriate secondary antibody (peroxidase-conjugated goat anti-rabbit IgG, 1∶1000; Beyotime, Hangzhou, China). Bound antibodies were visualized by the enhanced chemiluminescence detection system (Amersham Biosciences). Band intensities were quantified with Bandscan5.0 software.

### Pharmacological inhibitor treatment

The pharmacological reagents PI3K/Akt inhibitor LY294002 were obtained from Beyotime (Hangzhou, China) and were reconstituted in sterile DMSO and used at the (50 mM), DMSO at 0.1% concentration was used as the vehicle control. In all experiments with inhibitors, a tested concentration was used after careful titration experiments assessing the viability of the macrophages. The AM was treated with a given LY294002 for 1 h before stimulation by ST169 (H1N1), ST602 (H3N2) and HKG9 (H9N2).

### Statistical methods and data management

The results are presented as the mean ± SEM. The differences between groups were examined using Student's unpaired t-test. One-way ANOVA compared more than two groups, and post-hoc Newman-Keuls tests identified differences between groups. P<0.05 was considered to be significant.

## Results

### Preparation of AM

**Figure 1:**
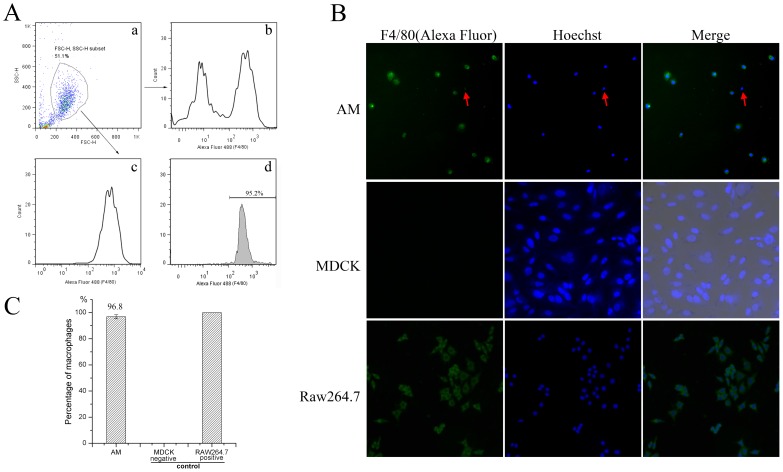
The Quantity and Purity of AM. (A) showed that AM was detected by flow cytometry, result (a) showed that AM (R1, framed region) is 51.1% of BAL cells. (b) and (c) showed AM is framed as Alexa Fluor 488 labeled F4/80 positive cells (d) showed AM was purified by adhere to the plate and the purity was detected as 95.2% by flow cytometry. (B) showed AM was purified by adhere to the coverslip, Alexa Fluor 488 labeled rabbit anti mouse F4/80 was used as the macrophage membrane marker, and the nuclear was stained by Hoechst, negative cells were red arrow headed; negative (MDCK cells) and positive (Raw264.7 cells) control was processed follow the same condition. (C) showed the purity from five independent microscope captures (of picture B) was detected as 96.8%.

Converging studies have shown that M1 and M2 macrophages are functionally polarized in response to microorganisms and host mediators. Here, we freshly purified AM to detect the polarization pattern induced by different influenza subtypes and to demonstrate the function of AM in acute lung injury induced by influenza virus. Flow cytometry showed that the F4/80 positive cells were AM (Figure 1A.a.b.c), and the total number of AM obtained from 36 mice is approximately 1.5×10^7^ cells. Then, AM was purified by adherence to a culture dish, and the purity was determined through two experiments. Immunofluorescence showed AM have a purity to be 96.8% (Figure 1B and C), red arrow headed the negative cells (Figure 1B); Figure 1A.d showed flow cytometer analysis result, in which AM has a purity of 95.2%, which is similar to the result of Figure 1B and C.

Some studies use other macrophage cells instead of AM in the respiratory system investigation, because they are easier to collect than AM. In contrast, some results showed that AM is essential for studies on the respiratory system [30]. In this study, we collected different macrophages to detect their response toward influenza infection, including AM, BMDM, PM and the Raw264.7 cell line. Figure S1 in File S1 showed the result of macrophage cells infected with ST169 (H1N1) after 8 hours, which indicated that other cells do not respond the same way as AM. PM cells have a similar but more severe response (M2b), whereas BMDM and the Raw264.7 cell line exhibited a completely different response. BMDM showed dramatic higher level of IL-10, MGL1 and CD163 (M2 markers), and slight higher level of iNOS and IL-6 (M1 markers), which indicated that BMDM has a potential M2c phenotype [11]. Compare with AM, Raw264.7 cell line showed a similar change of M1 markers, but the level of IL-10 is not elevated, which indicate a M1 phenotype, and IL-10 in Raw264.7 has elevated after 12 hours (data not shown), which indicate this cell line is insensitive compare with AM. Therefore, AM is necessary for studying the polarization pattern of macrophages infected by influenza virus.

### Dynamic polarization of AM induced by Virus for 4 hours, 8 hours and 24 hours

We plated AM on plastic, after 2 hours, non-adherent cells were washed, and then the influenza virus and control (the same volume of influenza virus culture medium, MEM containing 0.5 µg/ml TPCK-trypsin and 2% BSA fraction V, was added instead of virus) were added to investigate the polarization of AM. On 0 hours (before viruses or control was added), the polarization markers of AM has no statistical difference between control and virus groups (data not shown), indicate that non-infected AM of all groups have similar character.

**Figure 2:**
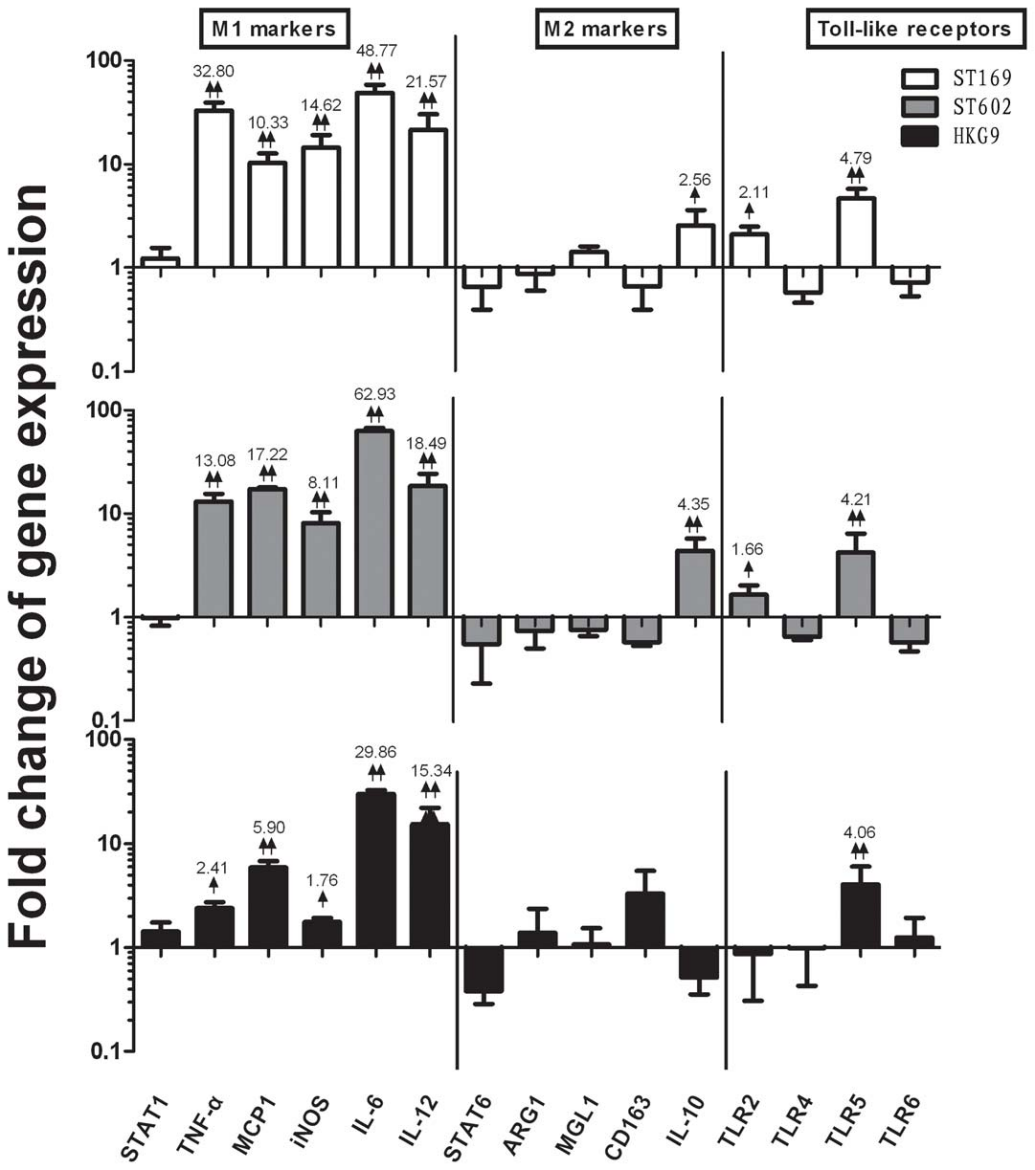
Gene expression of AM infected by influenza viruses at 2 MOI in vitro after 4 hours. Results are expressed as a ratio to mock-inoculated cells after 4 hours induction. ST169 (H1N1), ST602 (H3N2) and HKG9 (H9N2) promote M1 polarization of AM. mRNA levels of M1, M2 and Toll like receptors genes of AM was analyzed by qRT-PCR. All genes were normalized to GAPDH expression. Set as 1 and indicated by the horizontal X-axis, four duplication per gene was detected. ↑ =  mild upregulated (P<0.05), ↓ = mild downregulated (P<0.05); ↑↑ =  dramatically upregulated (p<0.01), ↓↓ = dramatically downregulated (P<0.01), the significant fold change were numbered. Three independent experiments have been processed.

Macrophages undergoing M1 or M2 polarization have characteristic profiles of marker expression. qRT-PCR test showed that after 4 hours of ST169 (H1N1) infection, compared with the 4 hours control, AM expressed dramatically higher levels of M1 markers, including TNF-α, MCP-1, iNOS, IL6 and IL-12. IL-10 (an M2 marker) was also up-regulated by 2.56-fold compared with the control. We infer that ST169 (H1N1) infection a M1 phenotype in the AM (Figure 2). ST602 (H3N2) induced a similar phonotype to ST169 (H1N1) in AM but with a slightly higher IL-10 expression (4.35-fold increased). Conversely, HKG9 (H9N2) induced dramatically higher levels of MCP-1 and IL-6, but lower levels of IL-10, which is compatible with a M1 phenotype. In addition, TLRs expression showed ST169 (H1N1) induced higher levels of TLR5 and TLR2 expression in AM, ST602 (H3N2) has the similar result, whereas HKG9 (H9N2) caused TLR5 expression up-regulation (Figure 2). When comparing the three influenza virus strains, we infer that the influenza virus induced AM to polarize to the M1 phenotype in the early stages (4 hours induction).

**Figure 3:**
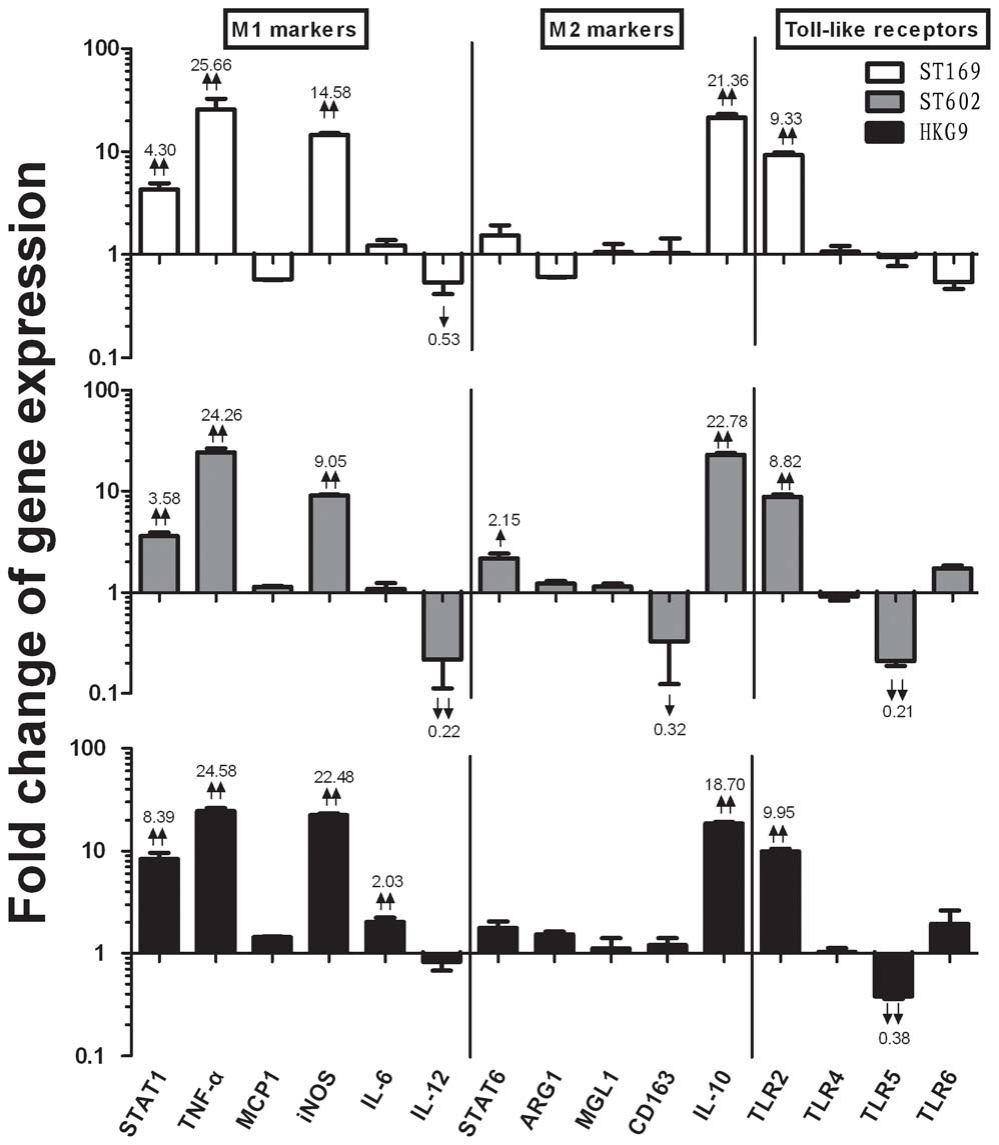
Gene expression of AM infected by influenza viruses at 2 MOI in vitro after 8 hours. Results are expressed as a ratio to mock-inoculated cells after 8 hours induction. Influenza viruses promote M2b polarization of AM, mRNA levels of M1, M2 and Toll like receptors genes of AM was analyzed by quantitative PCR. Set as 1 and indicated by the horizontal X axis, four duplication per gene was detected. ↑ =  mild upregulated (P<0.05), ↓ = mild downregulated (P<0.05); ↑↑ = dramatically upregulated (p<0.01), ↓↓ = dramatically downregulated (P<0.01), the significant fold change were numbered. Three independent experiments have been processed.

Figure 3 showed that, compared with the 8 hours control, three influenza virus strains induced AM to polarize toward the typical M2b phenotype. In this stage, all influenza viruses induced dramatically higher levels of TNF-α, STAT1 and iNOS, lower levels of IL-12 (M1 markers), and dramatically higher levels of the M2 marker IL-10. Our results showed that AM phenotypes were dynamically transferred from M1 to M2b after 8 hours induction. In addition, all three influenza virus strains induced TLR2 expression have also higher levels compared with the control.

**Figure 4:**
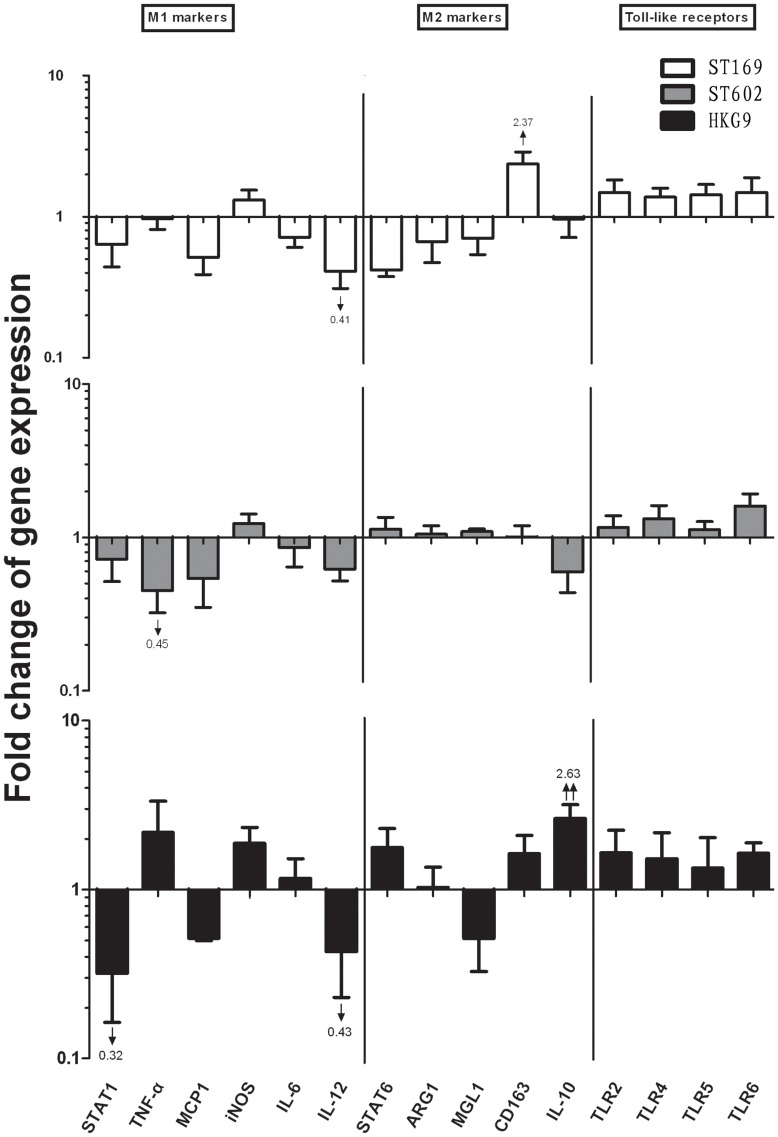
Gene expression of AM infected by influenza viruses in vitro at 2 MOI after 24 hours. Results are expressed as a ratio to mock-inoculated cells after 24 hours induction. ST169 (H1N1) and ST602 (H3N2) promoted immunosuppression of AM whereas HKG9 (H9N2) induced potential M2 pattern. mRNA levels of M1, M2 and Toll like receptors genes of AM was analyzed by qRT-PCR. Set as 1 and indicated by the horizontal X axis, four duplication per gene was detected. ↑ =  mild upregulated (P<0.05), ↓ = mild downregulated (P<0.05); ↑↑ = dramatically upregulated (p<0.01), ↓↓ = dramatically downregulated (P<0.01), the significant fold change were numbered. Three independent experiments have been processed.

After 24 hours of induction (Figure 4), some gene have slightly change, such as CD163 elevated by ST169 (H1N1) (2.37), and IL-10 elevated by HKG9 (H9N2) (2.63), whereas, most of the M1/M2 markers return to the normal level, it seems that influenza virus caused AM immunosuppression after 24 hours infection.

### Protein level of markers in AM polarization: TNF-α and IL-10 detected by Elisa and iNOS and Arg1 detected by Immunofluorescence

**Figure 5:**
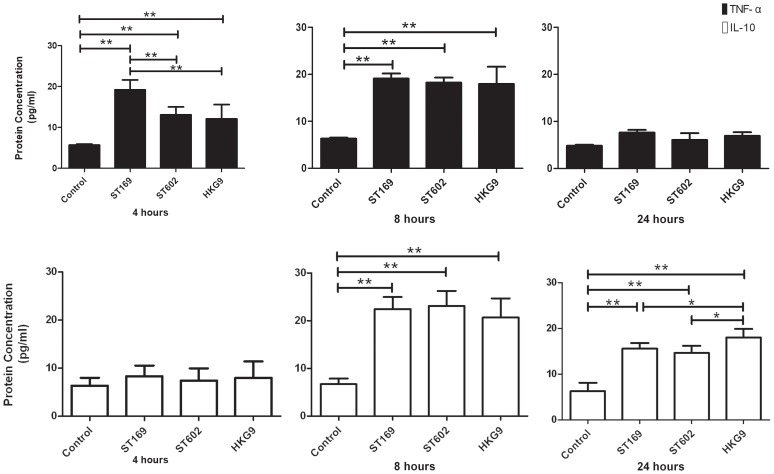
Elisa assay to detect the TNF-α and IL-10 expression. Cytokines in the AM culture supernatant were measured at the indicated times after influenza viruses infection at 2 MOI. (A) show the expression of TNF-α, (B) show the expression of IL-10. (C) indicate the trendline of the two cytokines expression. Experiments were performed in triplicate and data are expressed as the mean ± SEM, *p<0.05, **p<0.01. Three independent experiments have been processed.

ELISA analysis showed that TNF-α and IL-10 have a similar change to gene expression. Figure 5 indicated that after 4 hours of induction, the virus infection caused dramatically elevated TNF-α expression compared with the control (P<0.01). The induction by ST169 (H1N1) is stronger, while ST602 (H3N2) and HKG9 (H9N2) have a weaker induction. After 8 hours of induction, the viruses induced an up-regulation in TNF-α expression compared with the control, and there was no difference between influenza virus strains. Similar to the results of the gene expression, TNF-α expression was suppressed after 24 hours of induction by the virus.

IL-10 expression was also detected by ELISA analysis. Figure 5 showed virus induced up-regulation in the expression of IL-10 after 8 hours of induction compared with the control, and there was no difference between influenza virus strains. After 24 hours of induction, HKG9 (H9N2) induced higher IL-10 expression compare with the other two influenza virus strains.

**Figure 6:**
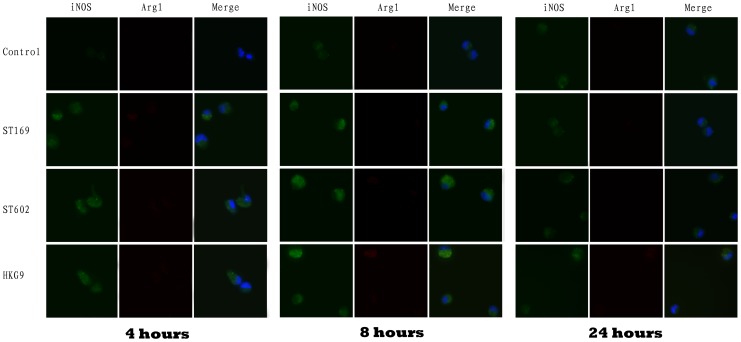
AM was infected by influenza viruses at 2 MOI, and immunostained for iNOS and Arg1 at the indicated times. The nucleus was stained with Hoechst (blue). Exposure to negative control (virus culture medium) resulted in an iNOS^low^Arg1^low^ phenotype at the three time pointes. Viruses infected AM induced iNOS (green) but not Arg1 (red) expression after 4 hours and 8 hours induction, after 24 hours induction, virus induced AM an iNOS^low^Arg1^low^ phenotype similar to negative control.

Immunofluorescence detection showed that after both 4 and 8 hours of virus challenge, the expression of iNOS was elevated; iNOS expression returned to control levels by 24 hours after induction (Figure 6). Arg1 expression was not notably changed after virus infection. The positive control (Figure S2 in File S1) was processed following Shikah Arora's method [31]. Protein level of markers in influenza virus induced AM polarization consistent with gene expression results. TNF-α^high^ iNOS^high^ IL-10^low^ Arg^low^ showed compatible with a M1 phenotype; whereas; TNF-α^high^ iNOS^high^ IL-10^high^ Arg^low^ showed compatible with a M2b phenotype; and all four proteins expression showed down-regulation after 24 hours induction.

### Influenza virus induce AM polarization via PI3K/Akt signaling pathway

**Figure 7:**
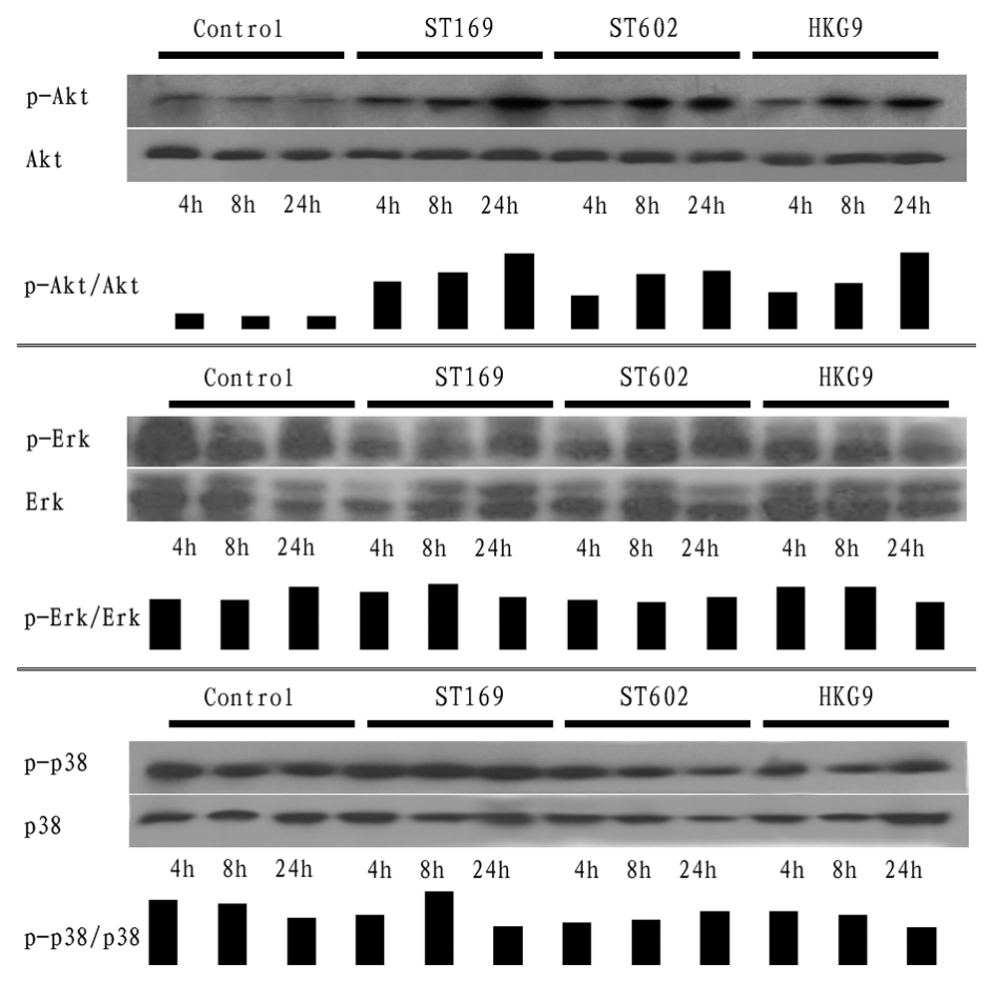
AM was infected by influenza viruses at 2 MOI, after 4 hours, 8 hours and 24 hours, cells were lysated and western was processed, the black bar denote the densitometric analysis of band intensity in western. Our result showed p-Akt was elevated after influenza virus infection, but not p-Erk or p-p38.

Our western blotting results demonstrated that PI3K/Akt pathway associated with influenza virus induced AM polarization. Analysis of phospho-Akt, phospho-ERK1/2, and phospho-p38 expression revealed that exposure of AM to ST169 (H1N1), ST602 (H3N2) and HKG9 (H9N2) showed notably increased phospho-Akt, but not phospho-Erk1/2 or phospho-p38 level, as compared with control (Figure 7).

**Figure 8:**
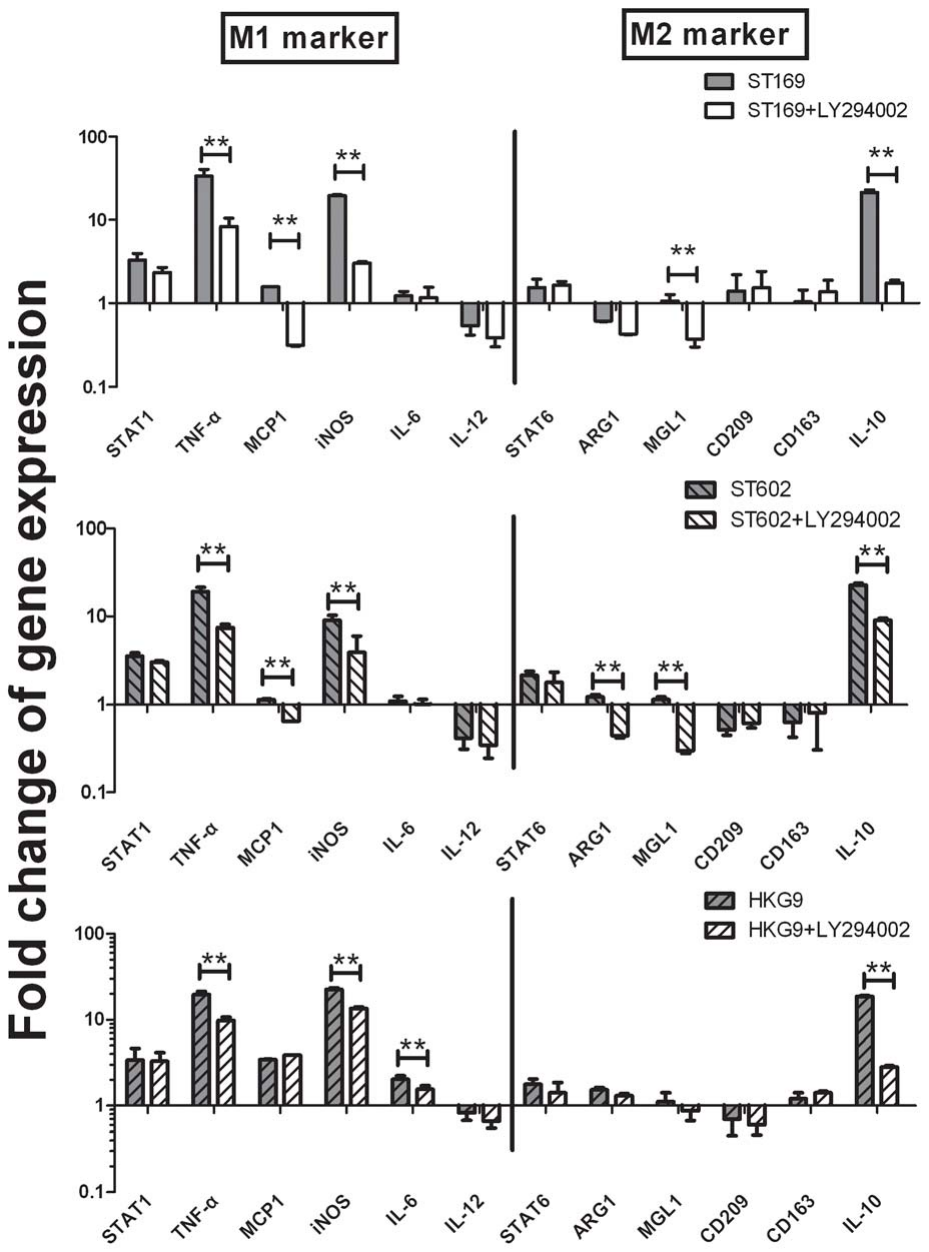
AM was treated with a given PI3K/Akt inhibitor, LY294002, for 1 h before stimulation by ST169 (H1N1), ST602 (H3N2) and HKG9 (H9N2). The LY294002 remarkable down regulate the macrophage polarization markers, such as, TNF-α, iNOS, MCP1 and IL-10. *p<0.05, **p<0.01.

Subsequently, the inhibitor of PI3K/Akt, LY294002, was added to conform whether influenza virus induce AM polarization via PI3K/Akt. Our qRT-PCR results showed remarkable decreased gene production of TNF-α, iNOS, MCP1 and IL-10, the macrophage polarization markers were dramatically down-regulated, but not be suppressed completely (Figure 8), which indicate PI3K/Akt signaling pathway is involved in influenza virus induce AM polarization, but not the unique way.

## Discussion

Macrophages from different tissues exhibit different phenotypes. In this study, we detected cells from four sources and confirmed that AM was the best cells to be used in this study. Monocyte-derived macrophages (MDM) were also used to study macrophage function; however, Sergij Goerdt demonstrated that MDM and AM is phenotypically different cells. AM has a alternatively activated phenotype, whereas MDM cells have a classical activated phenotype [32]. Debby van Riel found that there are significant differences between MDM and AM infected with different subtypes of influenza virus [30]. In this study, we investigated the polarization of AM, PM, BMDM and Raw264.7 cell line, and find that AM is irreplaceable in this study. AM is the majority of BAL cells, Crowell RE claimed that AM is approximately 78% of BAL cells. The standard protocol recommends Cut the abdominal aorta beneath intestines to drain blood, to eliminate red blood cell accumulation in the alveolar region to have fewer red blood cell (RBC) in BAL fluid and interstitial lung tissues [33]. In our study, we did not get rid of RBC, because AM has to be collected in shortest time to keep AM viable, another reason is that RBC could not adhere to the plate, it will be washed when we purified the AM.

In the preliminary experiment, we detected the optimal influenza virus MOI for infect AM. AM has the particular character, most studies report productive infection of human MDM but not AM [30], [34], [35], [36], [37].Most influenza virus could infect and replicate in AM, but no infectious virus could be released [30], [37], [38], [39], [40]. At present, it remains unclear how productive infection of AM by influenza virus is impaired. However, detection of viral nucleoprotein (NP) in infected cells suggests a post-translational block during the assembly or release of virions [30], [37]. Particularly, there is discordant evidence as to whether H5N1 infection is productive in AM, and it remains unclear if H5N1 viruses display an increased or equivalent infection rate of AM compared with seasonal strains [30], [37]. In this study, we selected three influenza virus strains, ST169 (H1N1), ST602 (H3N2) or HKG9 (H9N2), processed a preliminary experiment, and found that MOI 2 have an efficient infection, and did not cause AM cytopathic effect (CPE) (Figure S3 4 5 in File S1). Our study demonstrated the AM polarization patterns which were induced by. All three viruses induced M1 polarization in early stages and M2b in the middle stage. M1 macrophages develop in response to concomitant stimulation with IFN-γ and microbial products, such as LPS [41]. M1 cells exhibit potent microbicidal properties and promote strong Th1 responses. M1 macrophages display widespread morphology depending on their tissue location. Functionally, M1 cells are characterized by enhanced endocytic functions and enhanced ability to kill intracellular pathogens. This increase in microbicidal activity is primarily mediated by the release of nitric oxide (NO) from L-arginine by virtue of iNOS activity, a macrophage specific IFN-γ-inducible isoform of NO synthase [42]. The expression of MCP-1 was up-regulated in M1 cells. MCP-1 recruits monocytes, memory T cells, and dendritic cells to the sites of inflammation produced by either tissue injury or infection [43]. Lehrer, R.I and colleagues reported that MCP-1, abundant in rabbit lung macrophages, may contribute to anti-viral defenses by mediating the direct inactivation of influenza virus A/WSN viruses [22].

Our qRT-PCR results showed that AM phenotypes were dynamically transferred from M1 to M2b after 8 hours induction, and the protein expressions have similar results. The immunofluorescence also gives evidence, differential iNOS/Arg1 ratios in macrophages are not only an indicator of differential M1 versus M2 polarization status but also provide a good explanation for the opposite trend in virus clearance from the lungs. The iNOS-Arg1 ratios correlate with the respective levels of pulmonary clearance/growth of influenza virus, which is a good indication of influenza virus clearance by macrophages [44]. Up-regulation of Arg1 decreases the synthesis of fungicidal nitric oxide by competing with inducible nitric oxide synthase (iNOS) for the substrate L-arginine [45]. Arginine metabolism is characterized by high levels of inducible nitric oxide synthase (iNOS; NOS2) in M1 macrophages, whereas the arginase pathway predominates in M2a and M2c polarized macrophages, which results in the generation of ornithine and polyamines.

In this study, we demonstrate that the influenza virus (ST169 (H1N1), ST602 (H3N2) and HKG9 (H9N2)) induced immunosuppression of AM after 24 hours of incubation. Abundant evidence reveals that heterologous infections are acute or chronic infectious diseases that often occur as a result of prior viral infection predisposing a person to a secondary bacterial superinfection [23], [46]. We have a hypothesis that superinfection, after human influenza virus infection, correlates with the immunosuppression of AM. In a previous report, avian influenza virus (H7N9) induced a longer TH1 phase than the other viruses, which correlates with the avian influenza virus causing severe acute lung inflammation [47].

In this study, we demonstrated the AM polarization induced by different influenza viruses, and found PI3K/Akt signaling pathway involved in influenza induced AM polarization. Macrophage cells polarized majorly through the PI3K/Akt, MAPK/Erk or p38 MAPK signaling pathways [48], [49]. Zhang claimed that macrophage phenotypic polarization switched from pro-inflammatory M2b to anti-inflammatory M2a via PI3K/Akt–ERK signaling activation [48], Alicia Arranz demonstrated that overexpression or silencing of miR-155 confirmed its central role in Akt isoform-dependent M1/M2 polarization of macrophages [50]. Our results showed that influenza virus infected AM result in notably increased phospho-Akt, but not phospho-Erk1/2 or phospho-p38 level, and inhibit of PI3K/Akt with LY294002 caused dramatically down-regulation of AM polarization markers. However, the markers were not suppressed completely, it seems that activation of PI3K/Akt signaling pathway is not the unique mechanism, but it still definite that PI3K/Akt involved in the AM polarization induced by influenza virus.

In 2011, Wilbur H. Chen and colleagues reported that pulmonary alternatively AM (M2) are induced during influenza infection and may contribute to the elicitation of hypersusceptibility to a secondary bacterial infection. Their result showed high IL-10 and high TNF-α expression, which is similar to our results. However, they examined gene expression in total lung tissue, which may not accurately represent the gene expression in macrophages specifically [51]. We performed qRT-PCR to detect the gene expression profiles in mouse AM infected with ST169 (H1N1), ST602 (H3N2), HKG9 (H9N2) and mock-infected control cells at 4, 8 and 24 hours post-infection in vitro. The detected genes include three sets, M1 markers, M2 markers and toll-like receptors. M1/M2 markers were used to detect the pattern of AM polarization, while toll-like receptors were used to determine the potential function of the polarized AM. TLRs play important roles in host resistance to infections. A prerequisite for an effective host defense is the recognition of pathogens; TLRs are involved in this first step. In this study, we detected the expression of TLR2, 4, 5 and 6, which are expressed on the surface of macrophages [52], [53]. There is up-regulation of TLR5 during the polarization of AM after 4 hours of induction by viruses. TLR5 responds more generally to flagellated bacteria, which result in the expression of pro-inflammatory cytokines [54]. It seems that the anti-flagellated bacterial activity of AM was elevated following influenza virus infection. Our study indicated that after 8 hours of induction with viruses, the expression of TLR2 was up-regulated. TLR2 mainly recognizes bacterial and fungal cell wall components, such as LPS and lipopeptides. Our results suggest that AM potentially has higher anti-bacterial and anti-fungal activity after 8 hours of infection by virus. Conversely, results showed that TLR2 is activated as a result of apoptosis of macrophages [55], [56]. We presume that higher level of TLR2 after 8 hours of virus infection might have contributed to the observed immunosuppression at 24 hours following virus infection in our study.

Collectively, this study demonstrate that AM induced in vitro by influenza virus strains ST169 (H1N1), ST602 (H3N2) and HKG9 (H9N2) polarize to M1 in the early stage and later present M2b phenotype; and prolonged infection caused AM immunosuppression. The mechanism investigation showed that the influenza virus caused AM polarization via PI3K/Akt signaling pathway. All virus strains elicited similar results. Further study is needed to demonstrate the differences between human and avian influenza strains in the induction of AM in vivo and the significance of AM polarization in disease recovery.

## Supporting Information

File S1 Contains the following files: **Table.S1 in File.S1** Primers used in this study, F is forward primer and R is reverse primer. **Figure.S1 in File.S1** Investigation of gene expression of four sources macrophage, alveolar macrophage (AM), bow marrow derived macrophage (BMDM), peritoneal macrophage (PM) and Raw264.7 cell line, infected by H1N1 for 8 hours. mRNA levels of M1 and M2 markers genes were analyzed by qRT-PCR. All genes were normalized to GAPDH expression. Set as 1 and indicated by the horizontal X-axis. This result indicate AM is necessary in studying the polarization of macrophages infected by influenza virus. **Figure.S2 in File.S1** The positive control of Immunofluorescence. Cells were transferred to glass slide, and incubated for an additional 24 h at 37°C and 5% CO_2_ in media with recombinant mouse cytokines IFN-γ (100 ng/ml), IL-4 (20 ng/ml). then cells were treated as described in our manuscript. **Figure.S3 in File.S1** Gene expression of viruses infected AM (MOI 8) after 4 hours. Results are expressed as a ratio to mock-inoculated cells after 4 hours induction. After 4 hours induction, MOI 8 have the similar result to MOI 2, ST169 (H1N1), ST602 (H3N2) and HKG9 (H9N2) promote M1 polarization of AM. mRNA levels of M1, M2 and Toll like receptors genes of AM were analyzed by qRT-PCR. All genes were normalized to GAPDH expression. Set as 1 and indicated by the horizontal X-axis, three duplication per gene was detected. ↑ =  mild upregulated (greater than 2 and less than 4), ↓ = mild downregulated (greater than 1/4 and less than 1/2); ↑↑ = dramatically upregulated (greater than 4), ↓↓ = dramatically downregulated (less than 1/4), the significant fold change were numbered. A single experiment was done to select the optimal MOI, so there is no SD or SEM. **Figure.S4 in File.S1** Gene expression of viruses infected AM (MOI 8) after 8 hours. Results are expressed as a ratio to mock-inoculated cells after 8 hours induction. Influenza viruses promote M2b polarization of AM, mRNA levels of M1, M2 and Toll like receptors genes of AM were analyzed by quantitative PCR. Set as 1 and indicated by the horizontal X axis, three duplication per gene was detected. ↑ =  mild upregulated (greater than 2 and less than 4), ↓ = mild downregulated (greater than 1/4 and less than 1/2); ↑↑ = dramatically upregulated (greater than 4), ↓↓ = dramatically downregulated (less than 1/4), the significant fold change were numbered. A single experiment was done to select the optimal MOI, so there is no SD or SEM. **Figure.S5 in File.S1** Gene expression of viruses infected AM (MOI 8) after 24 hours. Results are expressed as a ratio to mock-inoculated cells after 24 hours induction. After 24 hours, Influenza viruses caused CPE (cytopathic effect) of AM under microscope. mRNA levels of M1, M2 and Toll like receptors genes of AM were analyzed by qRT-PCR, and the gene expression dramatically down-regulated by CPE. Set as 1 and indicated by the horizontal X axis, three duplication per gene was detected. ↑ =  mild upregulated (greater than 2 and less than 4), ↓ = mild downregulated (greater than 1/4 and less than 1/2); ↑↑ = dramatically upregulated (greater than 4), ↓↓ = dramatically downregulated (less than 1/4), the significant fold change were numbered. A single experiment was done to select the optimal MOI, so there is no SD or SEM.
(DOC)
