# Supplementary material for: PI3K/Akt Signaling Pathway Modulates Influenza Virus Induced Mouse Alveolar Macrophage Polarization to M1/M2b
Source: PLoS One. 2014 Aug 8;9(8):e104506. doi: 10.1371/journal.pone.0104506 (PMC4126709; doi:10.1371/journal.pone.0104506)
Supplement: File S1 — Contains the following files: Table.S1 in File.S1 Primers used in this study, F is forward primer and R is reverse primer. Figure.S1 in File.S1 Investigation of gene expression of four sources macrophage, alveolar macrophage (AM), bow marrow derived macrophage (BMDM), peritoneal macrophage (PM) and Raw264.7 cell line, infected by H1N1 for 8 hours. mRNA levels of M1 and M2 markers genes were analyzed by qRT-PCR. All genes were normalized to GAPDH expression. Set as 1 and indicated by the horizontal X-axis. This result indicate AM is necessary in studying the polarization of macrophages infected by influenza virus. Figure.S2 in File.S1 The positive control of Immunofluorescence. Cells were transferred to glass slide, and incubated for an additional 24 h at 37°C and 5% CO2 in media with recombinant mouse cytokines IFN-γ (100 ng/ml), IL-4 (20 ng/ml). then cells were treated as described in our manuscript. Figure.S3 in File.S1 Gene expression of viruses infected AM (MOI 8) after 4 hours. Results are expressed as a ratio to mock-inoculated cells after 4 hours induction. After 4 hours induction, MOI 8 have the similar result to MOI 2, ST169 (H1N1), ST602 (H3N2) and HKG9 (H9N2) promote M1 polarization of AM. mRNA levels of M1, M2 and Toll like receptors genes of AM were analyzed by qRT-PCR. All genes were normalized to GAPDH expression. Set as 1 and indicated by the horizontal X-axis, three duplication per gene was detected. ↑ = mild upregulated (greater than 2 and less than 4), ↓ = mild downregulated (greater than 1/4 and less than 1/2); ↑↑ = dramatically upregulated (greater than 4), ↓↓ = dramatically downregulated (less than 1/4), the significant fold change were numbered. A single experiment was done to select the optimal MOI, so there is no SD or SEM. Figure.S4 in File.S1 Gene expression of viruses infected AM (MOI 8) after 8 hours. Results are expressed as a ratio to mock-inoculated cells after 8 hours induction. Influenza viruses promote M2b polarization of AM, mRNA [file pone.0104506.s001.doc]

| GAPDH-F | GCATTGTGGAAGGGCTCA |
| --- | --- |
| GAPDH-R | AGGCGGCACGTCAGATC |
| STAT1-F | CGCCTTTGGGAAGTATTA |
| STAT1-R | CAGTTCGCTTAGGGTCGT |
| TNF-α-F | CTATGGCCCAGACCCTC |
| TNF-α-R | GCAGCCTTGTCCCTTGA |
| MCP1-F | CCTGCTGTTCACAGTTGC |
| MCP1-R | GTCTGGACCCATTCCTTC |
| iNOS-F | CCAAGCCCTCACCTACTTCC |
| iNOS-R | CTCTGAGGGCTGACACAAGG |
| IL-6-F | CCAGTTGCCTTCTTGGGACT |
| IL-6-R | GGTCTGTTGGGAGTGGTATCC |
| IL-12-F | GACCATCACTGTCAAAGAGTTTCTAGAT |
| IL12-R | AGGAAAGTCTTGTTTTTGAAATTTTTTAA |
| STAT6-F | GCCAAAGACCTGTCCATT |
| STAT6-R | CCATCTGTTCGGGCTTAT |
| ARG1-F | TGGCTTGCGAGACGTAGAC |
| ARG1-R | GCTCAGGTGAATCGGCCTTTT |
| MGL1-F | TGAGAAAGGCTTTAAGAACTGGG |
| MGL1-R | GACCACCTGTAGTGATGTGGG |
| CD209-F | CACTGCCTGCCACAAT |
| CD209-R | TTCCAGCCGTCATCTC |
| CD163-F | TCCACACGTCCAGAACAGTC |
| CD163-R | CCTTGGAAACAGAGACAGGC |
| IL-10-F | ATACTGCTAACCGACTCCT |
| IL-10-R | ATGGCCTTGTAGACACCT |
| TLR2-F | AGACGCTGGAGGTGTTGG |
| TLR2-R | AACGAAGCATCTGGGAGT |
| TLR4-F | AGAATGAGGACTGGGTGA |
| TLR4-R | TGTAGTGAAGGCAGAGGT |
| TLR5-F | GGCTCAACCAAACCAACG |
| TLR5-R | GGGTGATGACGAGGAATAG |
| TLR6-F | AACTCACCAGAGGTCCAA |
| TLR6-R | TCTTCCCTGTCGATTCTC |

Table.S1


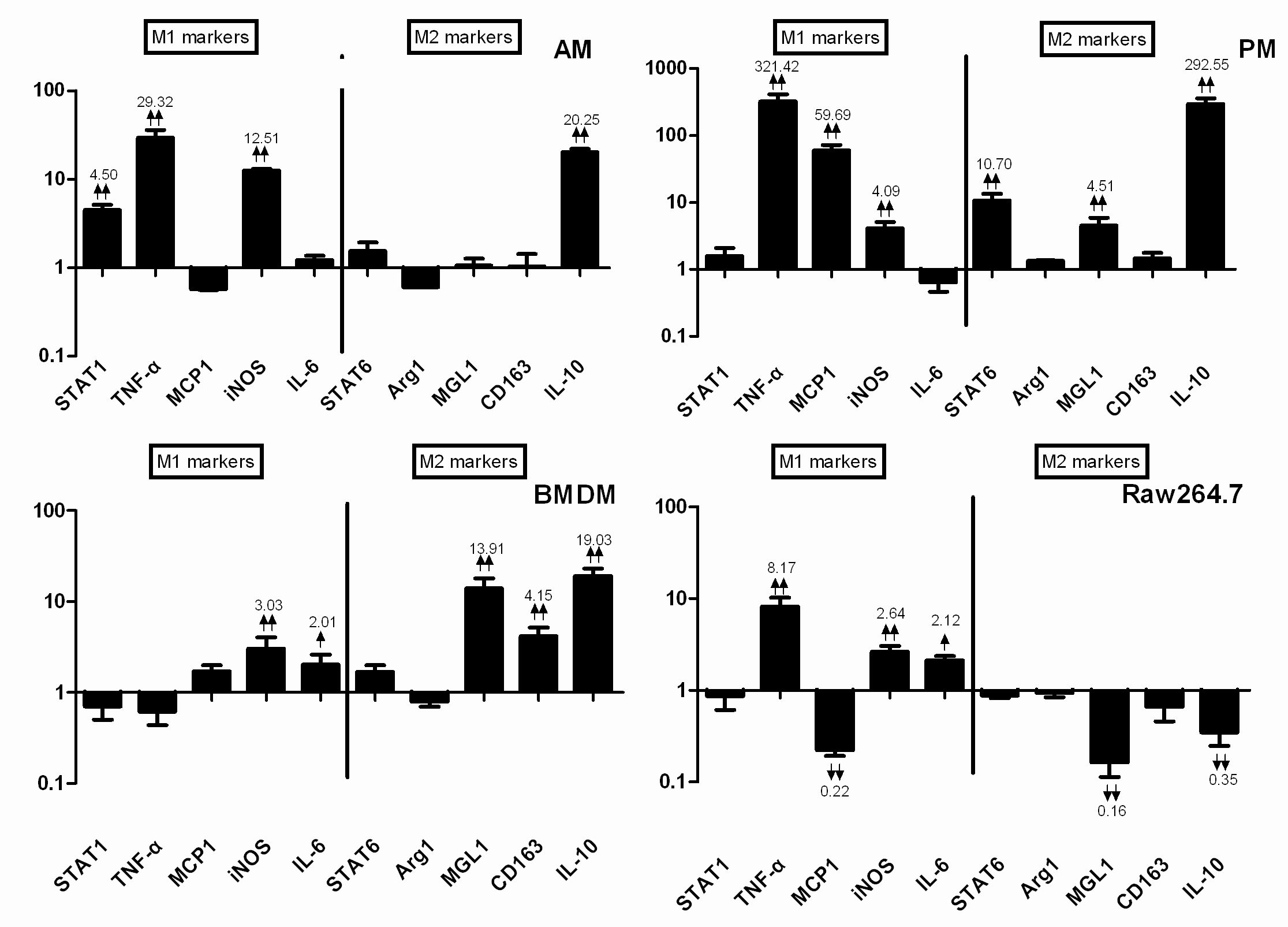


Figure.S1


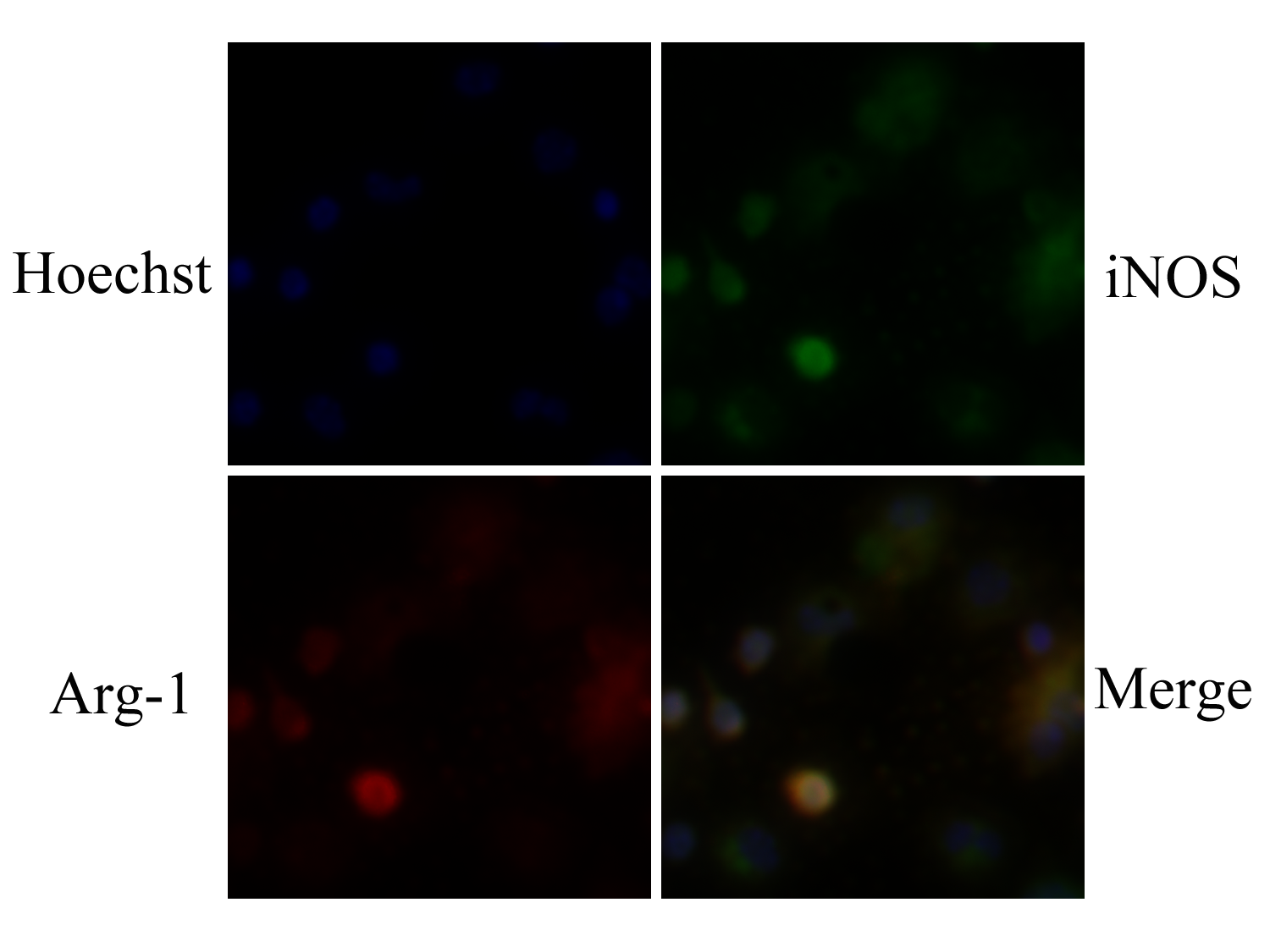


Figure.S2


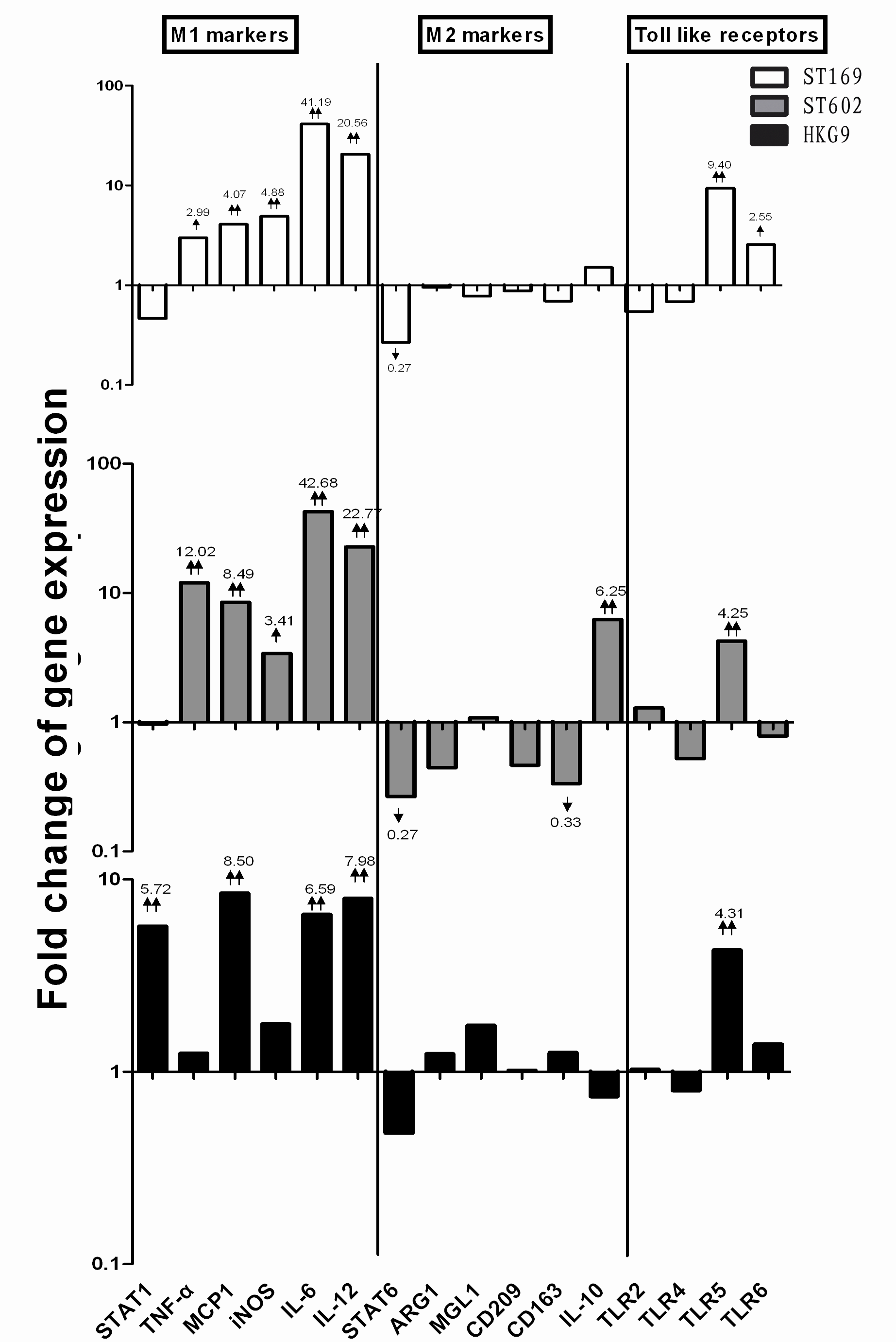


Figure.S3

**
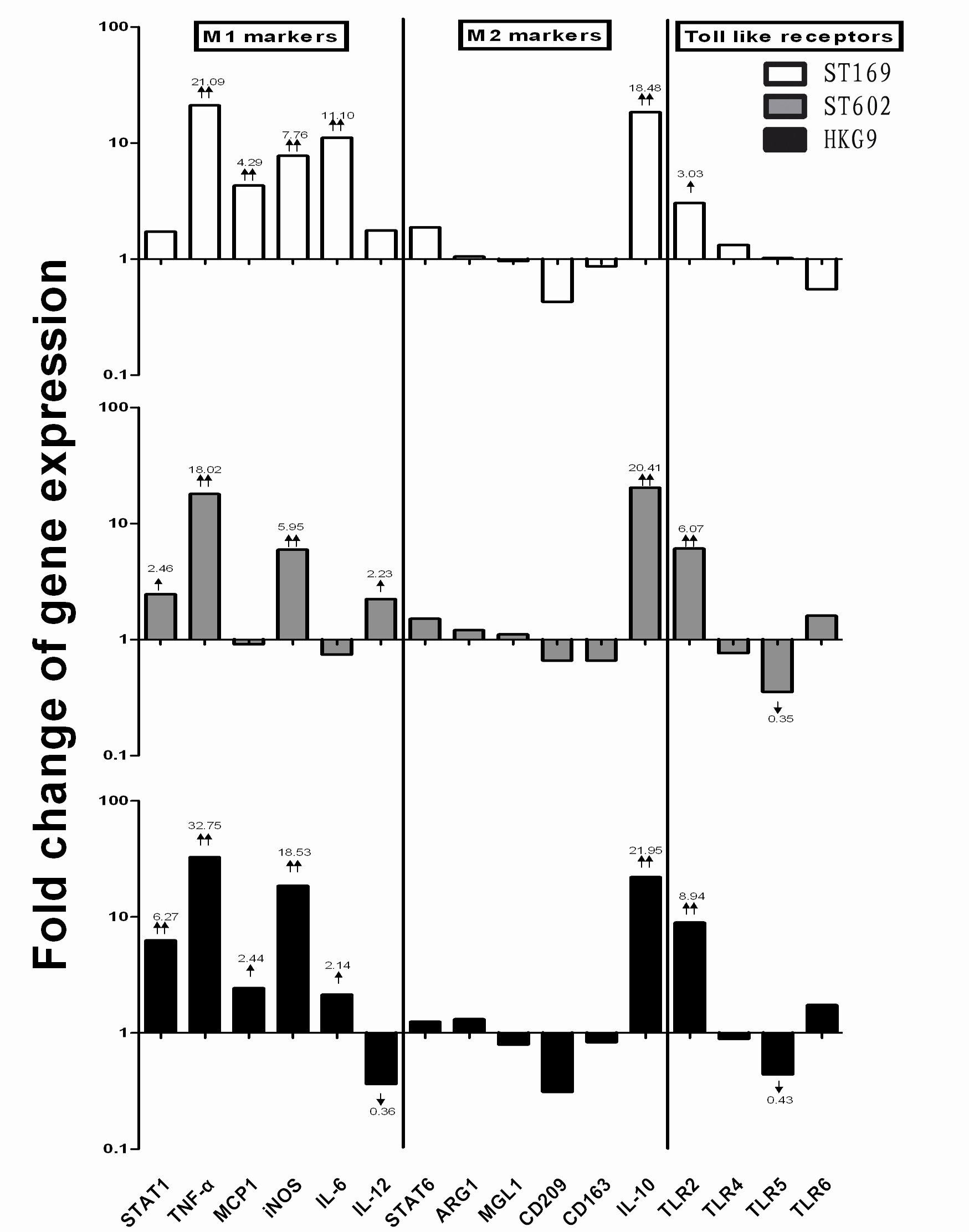
**

Figure.S4

**
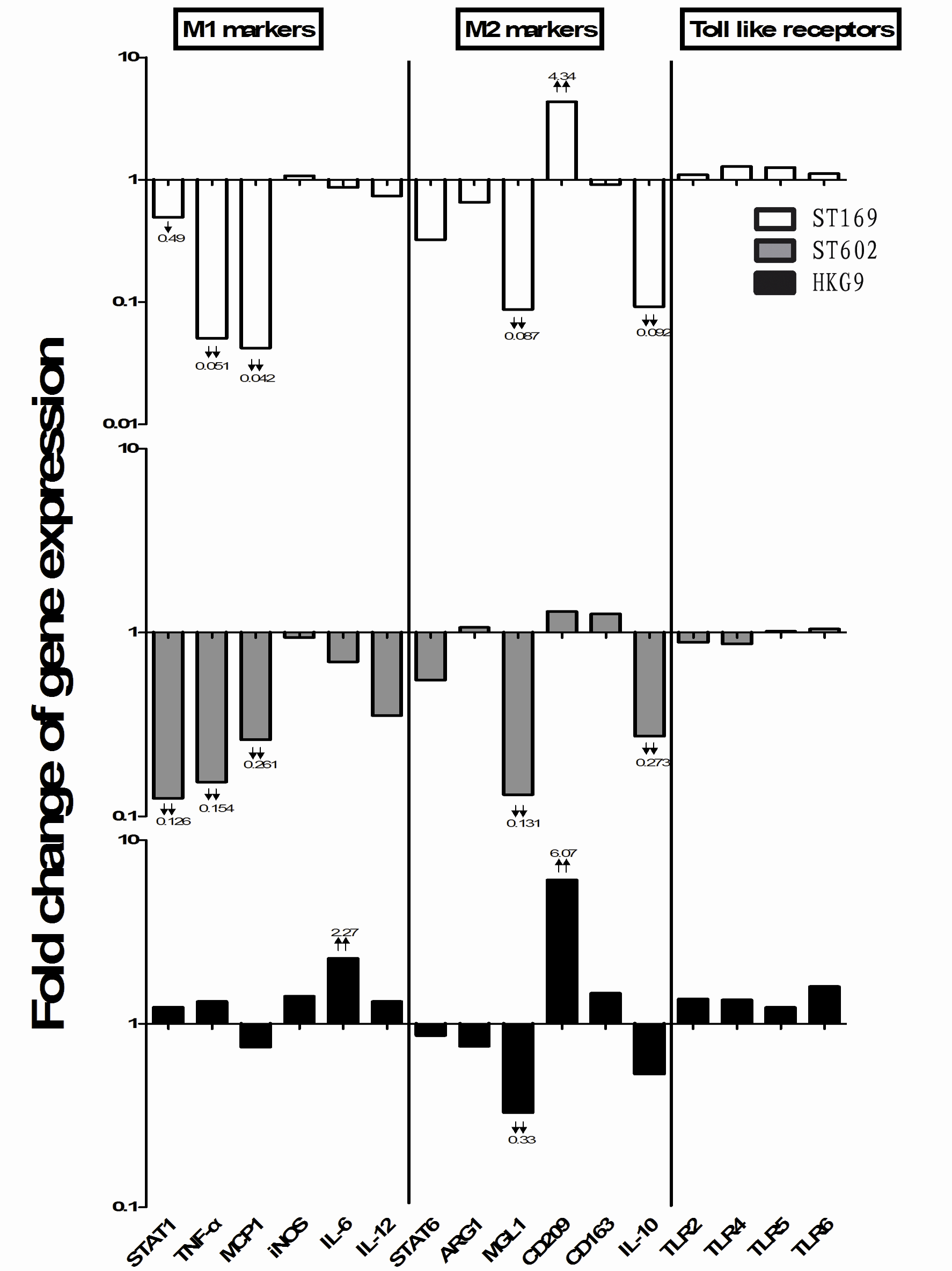
**

Figure.S5
